# Modelling the Bioaccumulation of Ciguatoxins in Parrotfish on the Great Barrier Reef Reveals Why Biomagnification Is Not a Property of Ciguatoxin Food Chains

**DOI:** 10.3390/toxins17080380

**Published:** 2025-07-30

**Authors:** Michael J. Holmes, Richard J. Lewis

**Affiliations:** Institute for Molecular Bioscience, The University of Queensland, Brisbane 4072, Australia; richard.lewis@imb.uq.edu.au

**Keywords:** ciguatera, ciguatoxin, *Gambierdiscus*, *Fukuyoa*, *Scarus*, *Chlorurus*, parrotfish, Great Barrier Reef, biomagnification

## Abstract

We adapt previously developed conceptual and numerical models of ciguateric food chains on the Great Barrier Reef, Australia, to model the bioaccumulation of ciguatoxins (CTXs) in parrotfish, the simplest food chain with only two trophic levels. Our model indicates that relatively low (1 cell/cm^2^) densities of *Gambierdiscus*/*Fukuyoa* species (hereafter collectively referred to as *Gambierdiscus*) producing known concentrations of CTX are unlikely to be a risk of producing ciguateric fishes on the Great Barrier Reef unless CTX can accumulate and be retained in parrotfish over many months. Cell densities on turf algae equivalent to 10 *Gambierdiscus*/cm^2^ producing known maximum concentrations of Pacific-CTX-4 (0.6 pg P-CTX-4/cell) are more difficult to assess but could be a risk. This cell density may be a higher risk for parrotfish than we previously suggested for production of ciguateric groupers (third-trophic-level predators) since second-trophic-level fishes can accumulate CTX loads without the subsequent losses that occur between trophic levels. Our analysis suggests that the ratios of parrotfish length-to-area grazed and weight-to-area grazed scale differently (allometrically), where the area grazed is a proxy for the number of *Gambierdiscus* consumed and hence proportional to toxin accumulation. Such scaling can help explain fish size–toxicity relationships within and between trophic levels for ciguateric fishes. Our modelling reveals that CTX bioaccumulates but does not necessarily biomagnify in food chains, with the relative enrichment and depletion rates of CTX varying with fish size and/or trophic level through an interplay of local and regional food chain influences. Our numerical model for the bioaccumulation and transfer of CTX across food chains helps conceptualize the development of ciguateric fishes by comparing scenarios that reveal limiting steps in producing ciguateric fish and focuses attention on the relative contributions from each part of the food chain rather than only on single components, such as CTX production.

## 1. Introduction

Ciguatera is a disease caused by eating normally edible tropical and subtropical fishes that have become contaminated through their diet with a class of potent, lipid-soluble toxins called ciguatoxins (CTXs) [1]. It is estimated to poison > 25,000 people annually [2], with ~500,000 people poisoned across the Pacific basin between 1973 and 2008 [3]. Ciguatoxins are produced by benthic dinoflagellates that belong to the genera *Gambierdiscus* and *Fukuyoa* [4]. Twenty species of *Gambierdiscus* and four species of *Fukuyoa* have so far been described [4,5,6,7,8], with eight *Gambierdiscus* and one *Fukuyoa* species so far confirmed from the east coast of Australia including the Great Barrier Reef [8,9]. Benthic dinoflagellates are typically found as epiphytes on turf or macroalgae on coral reefs although they can also be found on rocky reefs and other substrates [4]. Higher-trophic-level predatory fish become poisonous from feeding on herbivorous/detritivorous/grazing/scraping fish species (hereafter collectively referred to as herbivores) that accumulate CTX by feeding on turf or macroalgae supporting CTX-producing populations of *Gambierdiscus* and/or *Fukuyoa* (reviewed by Holmes et al. [9]).

The structures of the dominant CTX analogs contaminating fish vary between the major oceans, with the Pacific Ocean-CTX (P-CTX) and Caribbean-CTX (C-CTX) being the best characterised [4,10,11,12,13]. The structures of the Indian Ocean-CTX (I-CTX) [14,15] have not yet been determined due to yield losses on purification. Two structural families of CTX dominate in the Pacific, P-CTX-1 (also known as CTX1B) and P-CTX3C (also known as CTX3C) [4]. P-CTX-1 is derived from oxidative biotransformation of P-CTX-4B (CTX4B) and/or -4A (CTX4A, 52-*epi*-CTX4B) produced by some species of *Gambierdiscus* and *Fukuyoa* (hereafter collectively referred to as *Gambierdiscus*) [4,16,17,18,19]. The biogeographical separation of different CTX analogs produced by various *Gambierdiscus* species is an active area of research [20], with P-CTX3C occurring in ciguateric fishes in the Indian Ocean [21,22] and I-CTX in the Pacific Ocean [23], and more recently the discovery of P-CTX-1, -2 (52-*epi*-54-deoxy-P-CTX-1), and -3 (54-deoxy-P-CTX-1) in fish from the southwestern Indian Ocean [22], although the lack of detection of I-CTX from these fish is puzzling. We have also suggested that fish contaminated with P-CTX will be found in the northeastern Indian Ocean [20]. Where possible, it is important to verify the origin of fish, as mislabeling and fraud can be common across global seafood markets [24,25].

Ciguatera along the east coast of Australia (and the Northern Territory) is caused by eating benthic and pelagic fishes contaminated with P-CTX-1 and its 54-dexoy analogs, P-CTX-2, and P-CTX-3 [26,27,28,29]. The other major family of CTX found throughout the Pacific basin are the P-CTX3C analogs [4], which have a different structural backbone (“E”-ring) to the P-CTX-1 congeners [18]. However, P-CTX3C analogs have not yet been detected from Australian fishes or benthic dinoflagellates. The evidence is that the P-CTX-4 to P-CTX-1 pathway dominates the marine food chains that cause ciguatera along the east coast of Australia (reviewed by Holmes et al. [9]).

Holmes et al. [9] developed conceptual models for the food chain transfer of P-CTX-4 from *Gambierdiscus* into intermediate vectors, and then to the higher-trophic-level fishes that typically cause ciguatera along the east coast of Australia. From these, we derived a simple numerical model to quantify these transfers in marine food chains into pelagic Spanish mackerel (*Scomberomorus commerson*) in Platypus Bay [30] and benthic grouper (*Plectropomus leopardus*) on the Great Barrier Reef [31]. These top-down models estimate the population densities of CTX-producing *Gambierdiscus* that produce the minimum CTX load to contaminate the flesh of high-trophic-level fishes with P-CTX-1 equivalents (eq.) that could cause mild poisoning in humans (0.1 μg P-CTX-1 eq./kg fish) [2]. This CTX concentration is 10-fold more than the precautionary limit recommended by the US FDA [32]. In contrast to our top-down model, Parsons et al. [33] developed a bottom-up model for the Caribbean for *Gambierdiscus* epiphytic on macrophytes and turf algae that incorporated seasonality, variable grazing, and *Gambierdiscus* taxa of varying toxicity. These complementary modelling approaches attempt to simulate and quantify the flow of CTX through marine food chains based upon limited experimental data. Improving these ecological models will likely be an iterative process of refining the kinetic functions that simulate toxin accumulation, transformation, and excretion in representative species that make up each step in the food chain, as well as the chemistry and kinetics for production of CTX analogs from species of *Gambierdiscus*. Our ecological model is currently based upon simple linear approximations that are unlikely to be fully representative of nature. However, our approach allows us to explore hypothetical scenarios and develop testable predictions about the ecology of ciguatera.

Parrotfishes (family Labridae (wrasses), subfamily Scarinae) are an abundant, diverse and ecologically important group of coral reef fishes with numerous species along the Great Barrier Reef [34]. Parrotfish are diurnal feeders that often spend > 90% of daylight hours feeding [35,36,37]. They are often categorised depending upon their functional mode of feeding as (1) browsers which primarily crop macroalgae, (2) excavators that bite and crush calcareous substratum with their beak-like jaws for cyanobacteria and other autotrophic organisms within the matrix, and (3) scrapers that scrape turf algae, cyanobacteria and associated detritus from the substratum (epilithic turf algal matrix) [38,39,40,41,42]. Herbivorous fishes, in particular parrotfishes and browsing surgeonfishes, are highly desired fishery targets across much of the Pacific [43,44,45,46,47], although not on the Great Barrier Reef [48,49] or parts of Fiji [50]. Parrotfish are a minor component of the catch of both commercial and recreational fishers on the Great Barrier Reef, with >16,000 fish apparently caught annually by recreational fishers across all Queensland waters [51], although anecdotal evidence suggests that some of these may be tusk fish misidentified as parrotfish (tusk fish *Choerodon* spp., carnivorous fishes also in family Labridae). As parrotfish are not targeted by commercial fishers, there is no specific category for them in the Queensland Government’s database (QFish) that records the catch and effort of commercial fishers [52], with parrotfish likely forming a minor component sold under the general category of “mixed reef fish”. Between 2008 and 2018 (inclusive), the average annual commercial catch of mixed reef fish from the Great Barrier Reef was 17 ± 7 tonnes (mean ± standard deviation) [52]. In comparison, the major targeted commercial fishery on the Great Barrier Reef is for coral trout (groupers) belonging to species of *Plectropomus,* with the average annual catch between 2008 and 2018 (inclusive) being 851 ± 133 tonnes/year [52].

Both scraper and excavator species of parrotfish have been implicated in causing ciguatera across Polynesia and Micronesia in the Pacific Ocean (summarized in Table S5 of Perkins et al. [53]), although we are not aware of any cases from Australia, including the Great Barrier Reef. This contrasts with carnivorous wrasses (Tripletail Maori wrasse, *Cheilinus trilobatus* and Venus tuskfish, *Choerodon venustus*) that have caused several ciguatera outbreaks from fish caught in waters off Queensland [1,54]. Less-polar CTXs initially named scaritoxins were first isolated from the steephead parrotfish *Chlorurus microrhinos* (formally attributed to *Scarus gibbus*) from French Polynesia [55,56] with the toxins later identified as analogs of P-CTX-4 [17,18]. Our model is based upon parrotfish (scrapers and excavators) grazing substrates supporting benthic populations of *Gambierdiscus* producing analogs of P-CTX-4 which are then transferred and bio-converted to P-CTX-1 to produce a fish whose flesh accumulates the modelled target concentration of 0.1 μg P-CTX-1 eq./kg. If parrotfish (scrapers and excavators) are targeting microorganisms for nutrition [38,41,42,57], then the consumption of epiphytic benthic dinoflagellates on the turf algae they feed on is likely high.

In this paper we adapt our numerical model for transfer of CTX across three [31] and four trophic levels [30] to the bioaccumulation of CTX from *Gambierdiscus* epiphytic upon turf algae into scraping and excavating parrotfish species, a two-trophic-level food chain. We simulate the bioaccumulation of CTX into swarthy parrotfish *Scarus niger* (supported by data for *S. tricolor* and *S. frenatus*) as a model for scraper species because the biometric data is available for these species in the literature, and we compare this with *Chlorurus* species, especially the steephead parrotfishes *C. strongylocephalus* and *C. microrhinos* as models for excavators. *Scarus niger* and *S. frenatus* are medium-bodied scrapers (maximum lengths 40 and 47 cm, respectively) and *C. microrhinos* is a larger-bodied excavator (maximum length 80 cm) common to both mid-shelf and outer reefs of the Great Barrier Reef [34,58]. *Chlorurus strongylocephalus* (maximum length 70 cm) and *S. tricolor* (maximum length 40 cm) are common to the Indian Ocean [40,58] but we include them in our comparisons because of the available biometric data in the literature. We initially model the bioaccumulation of P-CTX-4 into 25 cm (total length) *S. niger* and *C. strongylocephalus* as this corresponds to the minimum legal length (as of 2025) for the capture of all parrotfish species in Queensland. We subsequently compare relationships between fish length, weight, and modelled CTX loads for scraper and excavator species to examine the relative enrichment/dilution of CTX within and across trophic levels and whether this supports the suggestion that CTX biomagnifies across trophic levels.

## 2. Results and Discussion

### 2.1. Construction of a Model for Production of Ciguatoxic Parrotfish on the Great Barrier Reef

We adapt our previous model framework [31] to the simplest food chain (two trophic levels) to analyse the development of ciguateric parrotfish on the Great Barrier Reef). This modelling became possible after species-specific equations were published for estimating the area grazed by parrotfish as a function of fish length [40]. This overcomes a major issue with modelling ≥ 2-trophic-level food chains, estimating the area/biomass of substrate supporting *Gambierdiscus* populations grazed by different-sized herbivores, the intermediate vectors for transfer of CTX to predatory fishes that are the preferred target for fishers on the Great Barrier Reef. Such data allows better estimates for the potential CTX load that can be accumulated and transferred to carnivorous fishes [9]. Our model is premised on the numerous vertebrate and invertebrate grazers on the Great Barrier Reef rapidly consuming the standing biomass of turf algae (<5–<28 days) [59,60,61] which limits the capacity for population growth of epiphytic *Gambierdiscus*.

Our previous three- and four-trophic-level models [30,31] function without knowing where in the ciguatera food chain P-CTX-4A and -4B are bio-converted to P-CTX-1. These models also assumed a 1:1 stochiometric conversion of P-CTX-4 to P-CTX-1 occurring between production in *Gambierdiscus* and transfer and incorporation into the flesh of a third- or fourth-trophic-level predator. Parrotfish consume P-CTX-4A and -4B from feeding on *Gambierdiscus*, with at least one of these analogs being transferred into the flesh apparently without biotransformation and becoming a major contributor of its toxicity, sufficient to poison people with two phases of symptoms that differentiate it from other ciguatera cases [17,55]. P-CTX-4B is the major isomer extracted from *Gambierdiscus* (*G. polynesiensis*) cultures with an average ratio of 4A:4B of 0.6 ± 0.1 (mean ± standard deviation, n = 12) [62,63] and a maximum combined concentration of 0.6 pg P-CTX3C eq./cell [62]. As the amount of each P-CTX-4 analog bio-converted to P-CTX-1 and then transferred to flesh is unknown, we model all P-CTX-4 transferred to muscle as being equivalent to P-CTX-1 (1:1 conversion). This is a worst-case scenario that almost certainly overestimates toxicity as P-CTX-4A is believed to have 1/5th the toxicity of P-CTX-1, and P-CTX-4B to have 1/20th the toxicity of P-CTX-1 [4].

Our model suggests that a small (just legal-sized) scraper or excavator parrotfish could consume sufficient CTX to become mildly poisonous after feeding for <1–10 days on turf algae supporting 100 *Gambierdiscus*/cm^2^ producing either 0.6 or 1.6 pg P-CTX-4/cell (Table 1). However, we believe that cell densities of 100 cells/cm^2^ are probably rare globally, including on the Great Barrier Reef. Maximum cell densities of ~10 cells/cm^2^ are likely more common but still at the high end of the range of those most frequently found on screen assays (0.1–10 cell/cm^2^), with the median density being ~1 cell/cm^2^ [64]. Our model indicates that densities of 10 *Gambierdiscus*/cm^2^ could produce a mildly poisonous fish in <1 month of feeding, but more likely if feeding on cells producing a hypothetical high 1.6 pg P-CTX-4/cell (Table 1). However, as benthic substrates likely support a mix of *Gambierdiscus* species with differing capacities to produce CTX [65], it is probable that real-world densities of 10 cells/cm^2^ are rarely capable of producing a poisonous parrotfish on the Great Barrier Reef. Given that turf algae are generally turned over in <1 month by the many species of herbivores that occur across the Great Barrier Reef [59,60,61], it is unlikely that a legal-sized (25 cm) parrotfish could accumulate sufficient CTX to become poisonous from feeding on turf algae supporting 1 *Gambierdiscus*/cm^2^ (Table 1).

Factors that could affect the capacity of parrotfish flesh to cause human poisoning will vary depending upon where in the cycle of toxin accumulation, biotransformation, and depuration fish are captured and eaten. The toxicity of the flesh will partly depend upon how much P-CTX-4 has been bio-converted to P-CTX-1 at the time when the fish is captured. As an average of 1.7 ± 0.4 more P-CTX-4B than P-CTX-4A is produced by *Gambierdiscus* (*G. polynesiensis*) [62,63], and P-CTX-4A and P-CTX-4B have 1/5th and 1/20th the toxicity of P-CTX-1, respectively [4], it follows that if these analogs are accumulated into parrotfish flesh at the same ratio as they are produced in *Gambierdiscus*, then the parrotfish flesh would have only 11% of the toxicity before biotransformation of the P-CTX-4 to P-CTX-1 (assuming 1:1 bioconversion for each of the two P-CTX-4 isomers to P-CTX-1).

Our model suggests that relatively low (1 cell/cm^2^) densities of *Gambierdiscus* species producing known concentrations of P-CTX-4 are unlikely to be a risk of producing ciguateric parrotfishes on the Great Barrier Reef, unless CTX can accumulate and be retained in parrotfish over months of feeding, whereas cell densities equivalent to 10 *Gambierdiscus*/cm^2^ could pose a risk as sufficient CTX could accumulate in <1 month of feeding by *S. niger* (Table 1). This contrasts with our previous conclusion that cell densities of ≤10 *Gambierdiscus*/cm^2^ are unlikely to pose a significant risk for production of average-sized groupers (*P. leopardus*) from the Great Barrier Reef [31]. This is because fish from the second trophic level accumulate CTX without the additional losses between trophic levels that occur during predation [66,67,68,69,70]. These models (two, three or four trophic levels) are based on the rapid production and accumulation of CTX loads because of the rapid (<1 month) turnover of turf algae on the Great Barrier Reef [59,60,61], which would likely reduce the capacity for *Gambierdiscus* to bloom, and the rapid depuration of CTX that can occur in some fish species [68,69]. The accumulation of CTX into surgeonfish from feeding on as few as two *G. polynesiensis*/cm^2^ has recently been suggested [8], although it was not determined if this was sufficient to produce a fish capable of poisoning people. To date, there are no reports of ciguatera from parrotfish from Australia, although this could be because parrotfishes are only a minor component of fish captured and eaten from the Great Barrier Reef, or because of rapid depuration of CTX [68,69], or other unknown factors. In Pacific communities where coral reef fisheries have been exploited to levels that reduce grazing of turf algae on reefs, it is possible that higher densities of *Gambierdiscus* could have the time to bloom more often, resulting in production of ciguateric parrotfishes.

### 2.2. Biomagnification Is Not a Property of the Ciguatoxin Food Chains

Ciguatoxins are sometimes described in the literature as biomagnifying/cumulative in food chains [53,71,72,73] but what is meant by this is not always clear. Part of the confusion arises because biomagnification can encompass

- Differences in relative toxicity between fishes of sequential trophic levels (e.g., herbivore prey and carnivorous predator);
- Differences in toxicity between different sized fish of the same species within the same trophic level;
- Differences in toxicity between different sized fish of different species, but within the same trophic level/guild.

#### 2.2.1. Differences in Relative Toxicity Between Fishes of Sequential Trophic Levels (e.g., Herbivore Prey and Carnivorous Predator)

Unfortunately, stating that a compound biomagnifies can imply that this is a property of the toxin in the food chain, similar to how the concentration of hydrophobic organochlorine pesticides in higher trophic organisms can be greater than the concentration in their food [74]. We have previously shown how toxicity can increase or decrease across trophic levels depending upon the structural type of CTX in the food chain (P-CTX-1 or P-CTX3C), and whether the intermediate vectors in the food chain are feeding on high densities of CTX-producing *Gambierdiscus* for an extended period, or on a short-lived bloom [31]. That is, the circumstances within the food chain determine the flux of CTX and whether the predator trophic level is or is not enriched, relative to its herbivorous prey (either because the predator does not feed on sufficient CTX-contaminated prey or because of rapid depuration). These are not binary alternatives, but examples of different outcomes along a continuum of possibilities determined by the dynamics within local food chains.

Another factor that does not appear to have been addressed in considering differences in toxicity between predator and prey trophic levels is the size of the CTX-contaminated prey. Most predatory fishes eat their prey whole, which indicates that the range of potential prey sizes for a predator is from just large enough to be retained to being too large for the jaw apparatus [75]. The optimal size of prey is apparently 0.6 times the predator’s jaw width (mouth gape) [75], with prey size generally increasing with predator size [75,76,77,78]. A large predator can eat small or large-sized prey, whereas a smaller predator of the same or different species is more limited in the size of the prey they can consume. If CTX-contaminated prey were too large for smaller predators, this could favour larger predators being more toxic than smaller sizes of the same or different species, an effect unrelated to toxin biomagnification. The exceptions to this generalization would be if prey were distressed from consuming toxins that induced abnormal swimming behaviour [9,79] that induced attacks by multiple predators, or for predators that can cripple prey such as pelagic Scombrid mackerels, or rotational feeders such as muraenid (moray) eels [75]. Moray eels are high-trophic-level predators that bioaccumulate high CTX concentrations across ciguatera risk areas across the Pacific [13,73,80,81,82,83,84,85,86]. Rotational feeding (spinning) enables eels to tear apart prey too large to be swallowed whole, thus reducing constraints of gape limitation that characterize foraging in many predatory fishes [87]. It would be interesting if this method of feeding contributed to a greater accumulation of CTX concentrations in moray eels.

#### 2.2.2. Differences in Toxicity Between Different-Sized Fish of the Same Species Within the Same Trophic Level

**Figure 1:**
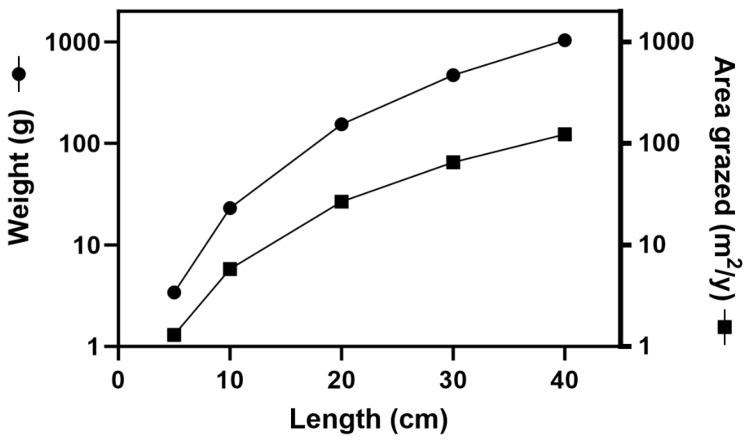
Relationships between total length, weight, and area grazed, for *Scarus niger*.

We examine differences in toxicity by comparing two different-sized parrotfish of the same species, one twice the length of the other, grazing for the same time over the same substrate supporting identical *Gambierdiscus* cell densities producing identical CTX concentrations. This results in the larger fish of the same species consuming 3.1-5.5-fold more CTX than the smaller, but this toxin load is distributed into 8.1–8.6-fold greater body weight than the smaller fish (Table 2 and Table 3). Assuming the rates of CTX biotransformation, transfer between tissues, and depuration are proportional between the two fish, this would result in the ingested CTX concentration being effectively diluted by the larger mass relative to the smaller fish, consistent with reports of an absence of any obvious relationship between toxicity and size within the same species [67,82,88,89,90,91,92,93,94], and with toxicity reducing with increasing size of herbivorous fish [67,89,93]. Therefore, if the different (allometric) scaling of length, weight, and area grazed (food intake) for *S. niger* (Figure 1, Table 4) is a general relationship for parrotfish and other herbivores, it could help explain the reduction in toxicity with increasing fish size for herbivores [67,89,93] with smaller fish bioaccumulating sufficient CTX to produce poisonous flesh over a shorter time (Table 4).

#### 2.2.3. Differences in Toxicity Between Different-Sized Fish of Different Species, but Within the Same Trophic Level/Guild

The differences in toxicity between different-sized fish of different species but within the same trophic level/guild depends upon how fish size scales relative to feeding capacity for that species. Comparing the modelled toxicity of the same-size fillet taken from a large scraper species (40 cm, *S. niger*) to a large excavator species (60 cm, *Chlorurus* spp./*C. microrhinos*) and assuming that both fish feed over the same time on the same *Gambierdiscus* density producing the same CTX concentration results in the fillet from the smaller fish being more toxic, even though the larger fish grazes a larger area, ingesting more *Gambierdiscus* and more CTX load (Table 5). However, this trend could depend upon the time taken for the toxin to be biotransformed and transferred into muscle for each species. Additionally, these inter-species comparisons must be used cautiously as the errors in the equations for estimating fish length, weight, and area grazed are likely different between species. However, our analysis provides a framework that explains some of the contradictory results previously reported for relationships between fish size and ciguatoxicity [83,89,90,93,95,96].

Overall, our analyses support previous conclusions that increasing fish size is not a good indicator of ciguatera risk [89,97]. In addition, as the underlying processes producing any relationship in fish size and toxicity are due to the interaction of factors occurring within and between trophic levels in local food chains, any such relationship could be subject to change over time. Therefore, it may be prudent to apply any relationships derived for size and toxicity cautiously for the management of ciguatera, at least without a better understanding of what drives toxicity in local food chains. Prohibiting the harvesting of larger-sized fish [98,99,100,101] does introduce a precautionary element into the management of ciguatera as larger poisonous fish can poison more people than an equally poisonous smaller fish, because more fillets can be processed from the larger fish [9]. Such a prohibition may also have unintended ecological and fishery advantages if the larger fish can be returned unharmed to the water (without barotrauma), as larger fish can contribute disproportionally more offspring to future generations [102].

### 2.3. Influence of Prey Size on Bioaccumulation of CTX into Predators

The ability to estimate the area grazed by different-sized parrotfish offers the opportunity to explore the impact of prey size on the bioaccumulation of CTX into the third-trophic-level fishes that are often the target species for fishers. This is an adaption of our model for production of toxic groupers (common coral trout, *Plectropomus leopardus*) on the Great Barrier Reef [31]. In this scenario we compare the toxin load accumulated by groupers preying upon juvenile parrotfish as a source of CTX, with the same size grouper hypothetically feeding on either of the following:

- One 10 cm parrotfish (23 g total weight) [103];
- An equivalent weight of 5 cm parrotfish (3.4 g total weight each fish) [103] = ~7 fish, where the different-sized parrotfish have fed over the same density of *Gambierdiscus* producing the same CTX concentration.

In this scenario, the smaller (5 cm) parrotfishes together ingest more CTX than the single larger parrotfish (Table 6). A predator, such as a grouper, feeding on an equivalent weight of parrotfish prey (one 10 cm fish, or seven 5 cm fish) could ingest ~50% more CTX from eating an equivalent weight of the smaller prey (Table 6). This suggests that the size and feeding history of prey are critical factors in the development of the CTX load in prey fish, and subsequently in the higher-trophic-level fish preying on them. This scenario could simulate groupers feeding repeatedly on juveniles as many parrotfish and surgeonfish species spawn repeatedly during the year [104], with cohorts of early life stages and juveniles suffering higher natural mortality relative to adults [105,106,107]. However, very small (post-settlement) parrotfish up to ~3 cm can have a more omnivorous diet including crustaceans and foraminifera that may limit the direct ingestion of *Gambierdiscus* for these early life stages [108]. Parrotfish are a common component of the diet of groupers (*Plectropomus*) on the Great Barrier Reef [76,109,110,111] and although prey size generally increases with predator size, all sizes of *P. leopardus* also consume small prey [76]. As we have previously suggested, the generally higher natural mortality suffered by juvenile fishes in the wild may facilitate the transfer of CTX through reef food chains by younger fish [9]. Mass recruitment events have been observed on some Pacific reefs for surgeonfishes [112,113,114], which could facilitate the trophic transfer of CTX through large numbers of juvenile herbivores to opportunistic predators such as groupers. Large schools of recruits (juvenile fish) of up to 5000 surgeonfish were observed in American Samoa, which grazed over reefs in a short-lived pulse, with most being eaten by predators [114]. However, it is also possible that juvenile fishes rapidly depurate ciguatoxins [66,69], which could be a mechanism that limits the trophic transfer of CTX from juvenile fishes to their predators.

The potential difference in toxicity between parrotfishes of different sizes feeding over the same reef area may have implications for ciguatera outbreaks. On the Great Barrier Reef, spearfishers are the only group of fishers that possibly target parrotfish, although generally, groupers and some tropical snappers are considered more desirable. If spearfishers were to take a parrotfish, they would more likely take larger fish, as a just-legal (25 cm) fish would often be considered not worth the effort (too small to retrieve a worthwhile fillet after taking account of damage from the spear to the relatively soft flesh). Larger species such as steephead parrotfishes like *C. microrhinos* may be preferred than smaller *Scarus* species. The scaling of reduced toxicity with doubling in length, demonstrated in Table 6 for *S. niger*, also occurs for these larger *Chlorurus* species (Tables S1 and S2). In this case, selecting larger fish may sometimes reduce the relative risk of poisoning. However, the absolute risk will depend upon the feeding history of individual fishes as well as at what point in the cycle of toxin accumulation, biotransformation, and depuration the fish is captured. Unfortunately, this somewhat reduced initial risk of poisoning from larger fish could subsequently be offset by an enhanced risk of poisoning for recreational fishers (line and spear) eating repeat meals from the same fish, something that generally does not occur when eating seafood meals from restaurants or other commercial sources [9].

Parrotfish are a major component of many small-scale fisheries of island communities across the Pacific, with fish mostly captured by spearfishing (often at night) [43,45,46,115,116,117]. If fishing pressure drives overexploitation of larger-sized fishes, leading to an increasing harvest of smaller-sized individuals of herbivorous fish species, this could produce an increased risk of poisoning for the communities relying upon these resources.

## 3. Summary and Conclusions

Our model indicates that relatively low (1 cell/cm^2^) densities of *Gambierdiscus*/*Fukuyoa* species epiphytic upon turf algae producing known concentrations of P-CTX-4 are unlikely to be a risk of producing ciguateric parrotfishes on the Great Barrier Reef. Cell densities of 10 *Gambierdiscus*/cm^2^ producing known maximum concentrations of analogs of CTX (0.6 pg Pacific-CTX-4/cell) are more difficult to assess but could be a risk. This contrasts with our previous conclusion that cell densities of ≤10 *Gambierdiscus*/cm^2^ are unlikely to pose a significant risk for production of average-sized coral trout from the Great Barrier Reef [31]. This is because eating fish from the second trophic level potentially risks consumption of CTX loads that accumulate without the subsequent losses that occur with transfer across additional trophic levels. Both model simulations (two or three/four trophic levels) are based on the rapid production and accumulation of CTX loads because of the mostly rapid turnover of turf algae on the Great Barrier Reef and may not apply to reef communities where fishery resources are exploited to levels that impact rates of grazing of algal substrates for *Gambierdiscus*. This rapid turnover (<1 month) of turf algae on the Great Barrier Reef likely limits the CTX load ingested by herbivores from feeding on the combination of cell density and CTX/cell sufficient to accumulate in fish flesh to cause human poisoning. Our model likely simulates the minimum loads that need to be accumulated to produce poisonous flesh as toxicity will be offset by depuration, although we have no data for depuration rates for parrotfish. All aspects of the model require more research to better define rates of toxin production, transfer across trophic levels, and depuration. Future development of the model should incorporate scenarios for depuration, especially where grazing is less likely to limit *Gambierdiscus* growth.

Our analyses are the first to demonstrate underlying mechanisms that can explain the observations of relative trophic dilution in ciguateric food chains and why biomagnification is not a property of ciguatoxin food chains. The allometric scaling of fish length-to-area grazed and weight-to-area grazed for parrotfishes suggests that larger fish of the same species can consume more CTX than smaller fish, but this toxin load is distributed into a greater body weight that effectively cause the toxin to be diluted by the larger body mass relative to the smaller fish. This is consistent with many reports of an absence of a general relationship between toxicity and size within the same species. Our food chain model helps focus attention on the relative contributions from the many factors leading to human poisoning and allows simulation of changes within food chains to develop testable hypotheses and predictions.

## 4. Material and Methods

**Figure 2:**
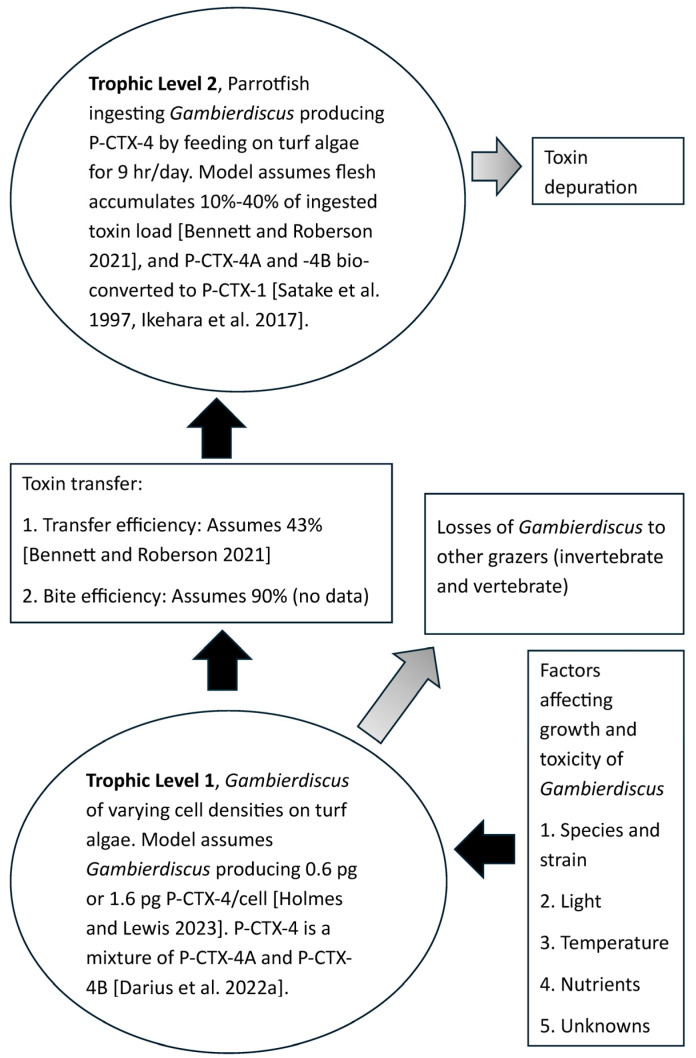
Model for the food chain transfer of P-CTX-4 from *Gambierdiscus* to parrotfish [17,18,31,62,70].

We adapt our conceptual and numerical models [9,30,31] to quantify the flow of ciguatoxins (CTXs) across two trophic levels of a hypothetical marine food chain on the Great Barrier Reef, Queensland, Australia, into parrotfish (Figure 2), principally *Scarus niger*, *Chlorurus microrhinos* and *C*. *strongylocephalus*, with data supplemented by life history characteristics from *S. tricolor* and *S. frenatus*. The major CTX that is known to contaminate fishes from the east coast of Australia including the Great Barrier Reef is Pacific-ciguatoxin-1 (P-CTX-1) [26], which is also known as CTX1B [4]. Our model starts with a target concentration of 0.1 μg/kg of P-CTX-1 equivalents (eq.) in the flesh of a parrotfish and then back-calculates the quantity of toxin required to be transferred to parrotfish to cause this level of contamination [30,31]. This target concentration is 10-fold higher than the US FDA-recommended limit of 0.01 μg P-CTX-1 eq./kg [32] and may cause mild poisoning in 2 out of 10 people [2]. For the P-CTX-1-family of toxins, the model incorporates the production of the less toxic P-CTX-1 precursors P-CTX-4A (CTX4A) and -4B (CTX4B) by *Gambierdiscus* and then the transfer and biotransformation of these in parrotfish. “CTX” is used throughout the paper to cover all toxic ciguatoxin precursors and metabolites and is estimated as P-CTX-1 or P-CTX-4 eq. We model scenarios for cell densities of 1, 10 and 100 *Gambierdiscus*/cm^2^ epiphytic on turf algae [30,31].

### 4.1. Model for Accumulation of P-CTX into Parrotfish

We use our model to calculate the number of days a parrotfish would need to feed on turf algae supporting various densities of *Gambierdiscus* producing different concentrations of P-CTX-4 to accumulate the target CTX concentration of 0.1 μg P-CTX-1 eq./kg flesh. The model is based on calculations for each trophic level (Figure 2) starting from back-calculation of the target CTX concentration in parrotfish, trophic level 2 (Table 7). The model parameters used to produce the CTX load in trophic level 1 consistent with the CTX target are calculated (Table 8) based upon the biometric data of the modelled parrotfish species (Table 9). The efficacy and limitations of the model are summarized in Table 10. Calculations for the model variables and assumptions (Table 7, Table 8 and Table 9) were performed using a commercial spreadsheet (Excel). Table 11 provides an example of the step-by-step calculations for one scenario, estimating the number of days that a 25 cm *S. niger* would need to feed on turf algae supporting 10 *Gambierdiscus*/cm^2^ producing 0.6 pg P-CTX-4/cell for the parrotfish to accumulate a flesh concentration of 0.1 μg P-CTX-1 eq./kg.

Graphs were constructed using GraphPad Prism 10.4.2. A major limitation of our model is that the uncertainties for the parameters and variables are unknown. It is possible that many variables and their error distributions will differ between species and geographic locations.

### 4.2. Background for Model Interpretation

The CTX load that accumulates into second-trophic-level herbivores is dependent on the CTX concentration of the *Gambierdiscus* they ingest, the number of cells ingested, and the time over which they are consumed. The number of cells ingested and the time taken to consume them are dependent on the density of cells on the substrate being consumed. The highest known concentration of P-CTX-4 is currently 0.6 pg P-CTX-4A and -4B eq./cell from cultures of a French Polynesian strain (RIK7) of *G. polynesiensis* isolated from the Gambier Islands [62]. We previously modelled *Gambierdiscus* producing 0.6 and 1.6 pg of P-CTX-4 eq./cell to produce mildly toxic Spanish mackerel and small groupers from the east coast of Australia [30,31], with an estimated 1.6 pg P-CTX-4 eq./cell based upon mouse bioassays of *Gambierdiscus* strains isolated from Platypus Bay and the Great Barrier Reef, Australia [30,31]. Such high concentrations of P-CTX-4 analogs are yet to be confirmed from any *Gambierdiscus* or *Fukuyoa* isolates, suggesting we are yet to develop culture conditions that match those found during toxic bloom events in the wild.

The density of *Gambierdiscus* that can occur on turf algae on the Great Barrier Reef is not known but likely varies with location and the turf species that form the three-dimensional matrix supporting benthic dinoflagellates. Thus, we base our models for turf algae on the *Gambierdiscus* densities reported from screen assays ([64,120] and references therein) with 9–12 species of *Gambierdiscus*, including *G. polynesiensis*, and 3 *Fukuyoa* species so far found on screens [121,122,123,124]. The densities of *Gambierdiscus* reported from screen assays is mostly due to migration from the water column with little from in situ cell growth, as only one cell generation could occur over the 24 h deployment of the screens [9]. This means that there must often be a considerable reservoir of *Gambierdiscus* in the water column near the benthos where the screen assays are conducted, possibly depending upon the species of *Gambierdiscus* present and the level of turbulence prevailing at the time [125]. However, to the best of our knowledge, the concentration and toxicity of this potential reservoir of cells in the water column near the benthos has not been quantified, but would likely be subject to considerable culling by the “wall-of-mouths” from benthic filter feeders and planktivorous fishes that can rapidly deplete the water column of plankton on coral reefs [126]. We recently suggested [20] that such plankton-based food chains may be a secondary route for accumulation of CTX after the detection of CTX in flying fishes [127]. As cell densities on screen assays plateau after ~24 h [64,120,128,129] it indicates that the screens are not conducive to growth and/or long-term attachment of *Gambierdiscus*, as settlement must be balanced by migration back into the water column. If settlement is not balanced by losses (migration/grazing), it is difficult to understand why the density on the screens would not increase (~double) every ~24 h from repeated settlement of cells out of the water column, at least over the range of densities known to occur on screens ~0.1–>100 cells/cm^2^ [64].

High screen assay densities of ≥100 cells/cm^2^ can occur [64] although it is not known if these reflect *Gambierdiscus* abundance on the benthos [130], such as on algal turfs. However, Yong et al. [121] did find the highest abundance of *Gambierdiscus* on screens in microhabitats where turf algae dominated. *Gambierdiscus* blooms can occur on relatively flat/low-relief surfaces [121,128,131,132,133] but these have generally not been quantified. Given that most cell densities on screens range between 0.1 and 10 cells/cm^2^ [64,120], we use screen densities of 1 and 10 cells/cm^2^ as starting points for our models of *Gambierdiscus* densities on turf algae, with 1 or 10 cells/cm^2^ theoretically producing > 100 cells/cm^2^ in <4 and <7 generations, respectively. That is, a fast population doubling time of 4 days (see Holmes et al. [9]) could produce > 100 cells/cm^2^ from 1 and 10 cells/cm^2^ in as little as 28 or 16 days, respectively. However, under the continuously variable environmental conditions in nature, maximal generation times are rarely likely to be sustained for the time it would take for a *Gambierdiscus* population on turf algae to reach 100 cells/cm^2^ from a starting density of 1 or 10 cells/cm^2^, with such sustained growth having to occur in the absence of population losses from grazing. Thus, we mostly limit interpretation of our model to ≤1 month because the rapid turnover of turf algae (<5–<28 days) by herbivores on the Great Barrier Reef [59,60,61] would likely reduce the opportunity for *Gambierdiscus* blooms. It is therefore likely that *Gambierdiscus* densities of ≥100 cells/cm^2^ on turf algae are rare. If all CTX-producing strains of *Gambierdiscus* respond similarly to *G. polynesiensis*, where the more potent strains have slower growth rates compared with less ciguatoxic strains [134], then even more time would be needed for production of high cell densities of highly toxic cells. The modelling of Parsons et al. [33] also suggests that there is often a lack of synchronicity between the various factors that must align for the trophic transfer of a CTX load sufficient to produce ciguateric fish.

We use conservative inputs for our model of the known transfer rates of CTX between trophic levels and the proportion of CTX load that distributes into fish flesh to cause poisoning. For some parameters, our conservative approach could underestimate the CTX load being accumulated by herbivores, but most would tend to overestimate the potential CTX load that could be accumulated (Table 10). On balance, we believe that our model likely overestimates the potential production and transfer of CTX between trophic levels, especially as our model assumes uniform cell densities of *Gambierdiscus* that all produce the same composition and concentrations of CTX. This is unlikely in nature as turf algae substrates are likely to support a mix of species [65] at spatially different cell densities, producing a range of CTX concentrations. However, cell densities composed of mixtures of low- and high-CTX-producing species could easily be incorporated into the model as more data becomes available. The modelling of monospecific densities producing uniform CTX concentrations provides useful worst-case scenarios.

## Figures and Tables

**Table 1:** Days required for a 25 cm parrotfish feeding on turf algae supporting *Gambierdiscus* producing either 0.6 pg or 1.6 pg P-CTX-4/cell to accumulate 0.1 μg P-CTX-1 eq./kg in its flesh.

	Days Feeding on Turf Algae to Accumulate 0.1 μg P-CTX-1 eq./kg in Flesh of Parrotfish			
*Gambierdiscus* Densities on Turf Algae (Cells/cm^2^)	25 cm (Total Length) *Scarus niger*, Medium-Bodied Scraper		25 cm (Total Length) *Chlorurus strongylocephalus*, Large-Bodied Excavator	
	0.6 pg P-CTX-4/*Gambierdiscus*	1.6 pg P-CTX-4/*Gambierdiscus*	0.6 pg P-CTX-4/*Gambierdiscus*	1.6 pg P-CTX-4/*Gambierdiscus*
1	144–575	54–216	286–1143	107–429
10	14–58	5–22	29–114	11–43
100	1–6	<1–2	3–11	1–4

**Table 2:** Ratios of fish weight and feeding area grazed for three medium-bodied scraper parrotfish (*Scarus* spp.) for fish of 20 cm and 40 cm total length.

		*Scarus niger*		*Scarus tricolor*		*Scarus frenatus*	
Fish	Total Length (cm)	Weight (g)	Area Grazed (m^2^/y)	Weight (g)	Area Grazed (m^2^/y)	Weight (g)	Area Grazed (m^2^/y)
A	20	211	27	208	21	226	47
B	40	1805	129	1724	65	1934	257
Ratio (B/A)	2.0	8.6	4.8	8.3	3.1	8.6	5.5

**Table 3:** Ratios of fish weights and feeding area grazed for large-bodied excavator parrotfish (*Chlorurus* spp.) for fish of 30 cm and 60 cm total length.

		*Chlorurus* spp. (Max Total Length 70–80 cm)	
Fish	Total Length (cm)	Weight (g) *Chlorurus* spp.—*C. microrhinos*	Area Grazed (m^2^/y) *C. strongylocephalus*
A	30	654–676	45
B	60	5266–5633	215
Ratio (B/A)	2.0	8.1–8.3	4.8

**Table 4:** Days required for a 10, 20, or 40 cm parrotfish (*Scarus niger*) to feed on turf algae supporting *Gambierdiscus* producing 0.6 pg P-CTX-4/cell to accumulate 0.1 μg P-CTX-1 eq./kg in its flesh.

	Days Feeding on Turf Algae Supporting *Gambierdiscus* Producing 0.6 pg P-CTX-4/Cell to Produce 0.1 μg P-CTX-1 eq./kg in Flesh of *Scarus niger* Parrotfish		
*Gambierdiscus* Densities on Turf Algae (cells/cm^2^)	10 cm (Total Length)	20 cm (Total Length)	40 cm (Total Length)
1	87–348	127–509	186–745
10	9–35	13–51	19–75
100	<1–4	1–5	2–7

**Table 5:** Comparison of modelled CTX accumulation into the flesh (μg P-CTX-1 eq./kg flesh) of smaller (*Scarus niger*) and larger-sized (*Chlorurus stronylocephalus*) species of parrotfish feeding on turf algae supporting *Gambierdiscus* producing 0.6 or 1.6 pg P-CTX-4 eq./cell over 30 days ^1^.

*Gambierdiscus* Densities on Turf Algae (cells/cm^2^)	40 cm (Total Length) *Scarus niger*, a Large Individual for this Scraper Species (Muscle Weight Assumed to be 42% of Total Weight = 436 g)		60 cm (Total Length) *Chlorurus strongylocephalus*, a Large Individual for This Excavator Species (Muscle Weight Assumed to be 42% of Total Weight = 2366 g)	
	0.6 pg P-CTX-4/*Gambierdiscus*	1.6 pg P-CTX-4/*Gambierdiscus*	0.6 pg P-CTX-4/*Gambierdiscus*	1.6 pg P-CTX-4/*Gambierdiscus*
1	0.01–0.04	0.03–0.10	<0.01–0.01	0.01–0.03
10	0.09–0.38	0.28–1.11	0.03–0.12	0.08–0.32

^1^ P-CTX-4 eq. presumed bio-converted to P-CTX-1.

**Table 6:** Comparing the CTX loads ingested by a model predator consuming 5 cm or 10 cm parrotfish (*Scarus niger*) that have fed on the same density of *Gambierdiscus*, producing the same concentration of P-CTX-4 eq./cell, over the same time.

	Parrotfish (*S. niger*) as Model Prey for Predatory Grouper		
Parrotfish	A (Small)	B (Large)	Ratio
Parrotfish total length (cm)	5	10	2 (B:A)
Weight (g)	3.4	23	6.8 (B:A)
Total load (ng) of P-CTX-1 eq. ingested by a single parrotfish feeding on 0.6 pg P-CTX-4/cell for 30 days at 10 *Gambierdiscus*/cm^2^	0.78	3.6	4.6 (B:A)
P-CTX-1 eq. load (ng) for equivalent weight of fish (23 g) = ~6.8 fish of 5 cm total length	5.3	3.6	1.5 (A:B)

**Table 7:** Model parameters for trophic level 2: parrotfish (*Scarus* or *Chlorurus* spp.) grazing turf algae supporting various densities of *Gambierdiscus* or *Fukuyoa* spp.

Variable	Model Values	Calculations, Assumptions, and Comments
Model target for P-CTX-1 concentration in flesh of parrotfish	0.1 μg P-CTX-1/kg	0.1 µg P-CTX-1/kg fish would likely cause mild poisoning in 2 out of 10 people [2] and is 10-fold more than the US FDA-recommended limit of 0.01 μg P-CTX-1 equivalents (eq.)/kg.
Flesh (fillet) recovery from parrotfish	42%	Median value of a range of meat recoveries for fillets (40–49%) taken from internet fishing sites for 5 species of *Scarus* spp.
Flesh (fillet) CTX burden	Range calculated as between 10 and 40% of the CTX load ingested by parrotfish	Flesh estimated to accumulate between 10 and 40% of the toxin load of the fish based upon Caribbean pinfish [70]. Clausing et al. [68] recently reported a slightly higher relative proportion of CTX retained in the muscle of surgeonfish (45%).
Parrotfish CTX load (μg)	Calculated depending upon fish weight (Table 9)	Based upon a 43% transfer rate (Table 8, [70]) and considering the P-CTX-4 analogs ingested (P-CTX-4A and -4B) are bio-converted to P-CTX-1 by the parrotfish. The ingested toxins are treated as P-CTX-1 eq.
Daily grazing rates (m^2^/d) for parrotfish on turf algae	Calculated from annual grazing rates (m^2^/y) depending upon species and fish total length (TL, cm)	Annual grazing rates (m^2^/y) calculated using equations derived by Lange et al. [40]: *S. niger* = 0.0367(TL^2.2^), *S. tricolor* = 0.1836(TL^1.591^), *S. frenatus* = 0.0138(TL^2.439^), *C. strongylopcephalus* = 0.0209(TL^2.256^)
The time parrotfish spend grazing on turf algae each day	9 h	Parrotfish are diurnal feeders that spend >90% of daylight hours feeding [35,36,37] and 9 h is consistent with the daily feeding times we used previously for surgeonfish on the Great Barrier Reef [31]. We have modified the daily feeding from 12 h used by Lange et al. [40] for parrotfish feeding close to the equator in the Maldives and Chagos Archipelago. However, feeding duration likely varies throughout the day, between seasons and with latitude
The efficacy of the parrotfish bite to remove and ingest *Gambierdiscus* from turf algae	90%	This rate is an assumption as there are no data available but is unlikely to be 100%. As scraping and excavator parrotfish are targeting microorganisms for nutrition [38,41,42,57] we assume the efficiency to be high

**Table 8:** Model parameters for trophic level 1: *Gambierdiscus* epiphytic upon turf algae grazed by parrotfish.

Variable	Model Values	Calculations, Assumptions, and Comments
The transfer rate for CTX between trophic level 1 and 2	43%	Based upon an average net CTX assimilation of 43% in pinfish [70]; also see Holmes and Lewis [30,31]. This term accounts for CTX losses between trophic levels. This transfer efficiency is similar to that reported for CTX from *G. polynesiensis* into mullet (42%, [66]). The actual transfer rates for the modelled species are not known
P-CTX-4 concentrations produced by *Gambierdiscus* consumed by parrotfish. These concentrations are varied depending upon the scenario being explored	0.6 pg or 1.6 pg P-CTX-4/cell	P-CTX-4 concentrations are assumed to be composed of a mix of P-CTX-4A and -4B; 0.6 pg P-CTX-4/cell is the maximum known concentration produced by a strain of *G. polynesiensis* from French Polynesia [62]; 1.6 pg/cell is a hypothetical concentration based upon mouse bioassay of *Gambierdiscus* strains isolated from Platypus Bay and the Great Barrier Reef, Australia [30,31]
*Gambierdiscus* densities on turf algae	0.1, 1, 10, 100, 1000 cells/cm^2^	Hypothetical (possible) cell densities of CTX-producing *Gambierdiscus* based upon ranges reported from 24 h benthic screen assays ([64] and references therein). We are not aware of any reports of cell densities ≥ 1000 cells/cm^2^; ~1 cell/cm^2^ is the median of a global range on screen assays [64]

**Table 9:** Length and weights of parrotfish species modelled. Minimum legal length for taking parrotfish in Queensland waters is 25 cm total length.

Scraping Species	Common/Local Name	Maximum Total Length ^1^ (cm)	Weight (g)–Total Length (TL, cm) Relationships	Reference for Weight–Length Relationships
*Scarus niger*	Swarthy parrotfish	40	Weight = 0.0411∙TL^2.7481^	[103]
*S. tricolor*	Tricolor parrotfish	40	FishBase calculator based upon geometric mean of 2 studies	[118]
*S. frenatus*	Sixband parrotfish	47	Weight = 0.0366∙TL^2.8162^	[103]
**Excavator species**				
*Chlorurus microrhinos*	Steephead parrotfish	80	Weight = 0.0174∙TL^3.07^	[119]
*C. strongylocephalus*	Steephead parrotfish	70	FishBase calculator based upon geometric mean of 5 studies	[118]

^1^ [58].

**Table 10:** Efficacy and limitations of model parameters.

Model Parameters for Two-Trophic-Level Food Chain	Description or Relevance	How Well Does the Model Parameter Simulate Reality?
Density of *Gambierdiscus* on turf algae	Actual densities not known but model explores an exponential range from 0.1 cells/cm^2^	Good, because model considers exponential range of possible densities. However, model does not account for finer-scale spatial or temporal factors that influence growth
*Gambierdiscus* species composition on turf algae	Model based upon monospecific composition of *Gambierdiscus* on turf algae eaten by parrotfish	Variable, as data suggests that sites can host multiple species [65]. Our model simulates worst-case scenarios (monospecific toxic blooms). Although this overestimates toxin production from mixed species assemblages on turf algae, it is useful to model toxic cell densities. The model could easily be adjusted for mixtures of species
*Gambierdiscus* CTX production	Highest known concentration [0.6 pg P-CTX-4/cell, 62] and a higher hypothetical concentration (1.6 pg P-CTX-4/cell)	Data-dependent. However, model does not account for environmental factors that influence toxin production. Model does not account for variation in toxicity of cells ingested by parrotfish
Grazing rates for parrotfish	Species-specific rates used from the literature	Data-dependent. Based upon published rates for area grazed/y [40]. Model does not account for seasonality affecting grazing rates or for schooling behaviour that can also affect grazing rates [37]
Parrotfish grazing (h/day)	Estimated average, consistent with previous modelling for the Great Barrier Reef [31]	Model adjusted from 12 h grazing/day for parrotfish near equator [40] to 9 h/day. Our model does not account for seasonality and latitude. Our model would underestimate ingested CTX if fish were grazing for up to 12 h/day
Grazing efficiency	Accounts for losses of material not ingested from the bite. Assumed 90%	No data, but as the fish are targeting microorganisms for nutrition, we assume the efficacy is high
Transfer efficiency of CTX between trophic levels	43%	Data-dependent, 43% [70]. Ledreux et al. [66] reported 42%. But rates for the species modelled not known
Bioconversion of P-CTX-4 to P-CTX-1 in parrotfish	Assumed 1:1 bioconversion from P-CTX-4 to P-CTX-1 to accumulate in muscle (fillets). We do not know or assume where in the fish that the bioconversion occurs. Conversion rates have relevance for the toxicity of the fillets consumed by people	Rates of bioconversion for P-CTX-4A and P-CTX-4B not known. Assuming a 1:1 bioconversion (P-CTX-1 eq.) our model likely overestimates the toxicity of the fillets
Biotransfer of CTX between parrotfish tissues, from gut to muscle. Model estimates between 10 and 40% of ingested load accumulates in muscle [70]	Losses occur with each toxin transfer, and it takes time for CTX to accumulate into muscle (fillets) [67,68,69]	Time for transfer between parrotfish tissues not known and not incorporated in model. Our model is based on an immediate transfer which could overestimate the toxicity of the fillets. Clausing et al. [68] reported 45% of CTX retained by muscle of surgeonfish. On this basis, our use of 10–40% could slightly underestimate the toxicity of muscle (fillets)
CTX load accumulated in parrotfish muscle (fillet)	Based upon CTX load ingested after losses during transfer and grazing efficiency	Worst-case scenario. On-going, possibly simultaneous rates of accumulation, bioconversion, and depuration not incorporated in model
Depuration of CTX from parrotfish muscle	Depuration is time-dependent so becomes more important the longer the duration explored in the model scenarios	Not included over the ~1 month we mostly limit model interpretation. Our model likely produces worst-case scenarios that overestimate the toxicity of muscle (fillets) because P-CTX-1, -2, and -3 have been suggested to depurate from groupers with half-life of ~1 month [69]. Additionally, only 26% of the P-CTX3C-load ingested was retained by surgeonfish after 4 months of feeding on *G. polynesiensis* [68], which suggests a faster depuration rate than Li et al. [69]

**Table 11:** Example scenario for back-calculating the number of days a 25 cm *Scarus niger* would need to feed on turf algae hosting 10 *Gambierdiscus*/cm^2^ producing 0.6 pg P-CTX-4/cell to produce a flesh concentration of 0.1 μg P-CTX-1 eq./kg.

Estimating the Number of *Gambierdiscus* to Produce a Flesh Concentration of 0.1 μg P-CTX-1 eq./kg in a 25 cm Parrotfish (*S. niger*)		
Calculating	Result of Calculation	Source/Reference for Calculation
Parrotfish weight for 25 cm fish	285 g	Table 9
Muscle (flesh) weight for 25 cm fish	120 g	Table 7
P-CTX load to produce a concentration of 0.1 μg P-CTX-1 eq./kg in 120 g flesh	1.2 × 10^−8^ g	
P-CTX load for the fish based upon flesh having 10% to 40% of toxin	3.0 × 10^−8^ to 1.2 × 10^−7^ g	Table 7
Number of *Gambierdiscus* producing 0.6 pg P-CTX-1 eq./cell to produce 3.0 × 10^−8^ to 1.2 × 10^−7^ g P-CTX-1 eq.	5.0 × 10^4^ to 2.0 × 10^5^ cells	
Number of *Gambierdiscus* producing 0.6 pg P-CTX-1 eq./cell to produce 3.0 × 10^−8^ to 1.2 × 10^−7^ g P-CTX-1 eq. incorporating an assimilation efficiency of 43% across trophic levels	1.2 × 10^5^ to 4.7 × 10^5^ cells	Table 8
**Estimating minimum number of days of feeding by *S. niger* to ingest 1.2 × 10^5^ to 4.7 × 10^5^ *Gambierdiscus***		
Area of turf algae scraped in 1 day (9 h) by parrotfish	897.2 cm^2^	Table 7
Number of *Gambierdiscus* ingested/day from turf algae with 10 *Gambierdiscus*/cm^2^	8972 cells	
Number of days to ingest 1.2 × 10^5^ to 4.7 × 10^5^ cells	12.9 to 51.8 days	
Number of days to ingest 1.2 × 10^5^ to 4.7 × 10^5^ cells adjusted for a 90% ingestion efficiency	14.4 to 57.5 days	Table 7
Model output	14 to 58 days (see Table 1)	

## Data Availability

The original contributions presented in this study are included in the article/Supplementary Material. Further inquiries can be directed to the corresponding author.

## Supplementary Materials

The following supporting information can be downloaded at: https://www.mdpi.com/article/10.3390/toxins17080380/s1, title; Table S1: Comparison of CTX ingested by 30 and 60 cm steephead parrotfish (*Chlorurus microrhinos*) feeding for 30 days on turf algae supporting 10 *Gambierdiscus*/cm^2^ producing either 0.6 or 1.6 pg P-CTX-1 eq./cell: Table S2: Comparison of CTX ingested by 30 and 60 cm steephead parrotfish (*Chlorurus microrhinos*) feeding for 30 days on turf algae supporting 10 *Gambierdiscus*/cm^2^ producing either 0.6 or 1.6 pg P-CTX-1 eq./cell (Table S1) but expressed per g of parrotfish.
