# Supplementary material for: Modelling the Bioaccumulation of Ciguatoxins in Parrotfish on the Great Barrier Reef Reveals Why Biomagnification Is Not a Property of Ciguatoxin Food Chains
Source: Toxins (Basel). 2025 Jul 30;17(8):380. doi: 10.3390/toxins17080380 (PMC12390024; doi:10.3390/toxins17080380)
Supplement: Supplementary file 1 [file toxins-17-00380-s001.zip › toxins-3733948-supplementary.pdf]

Supplementary Materials: Modelling the Bioaccumulation of Ciguatoxins in Parrotfish on the Great Barrier Reef Reveals Why Biomagnification Is Not a Property of Ciguatoxin Food Chains

Michael J. Holmes and Richard J. Lewis

Table S1. Comparison of CTX ingested by 30 and 60 cm steephead parrotfish (*Chlorurus microrhinos*) feeding for 30 days on turf algae supporting 10 *Gambierdiscus*/cm^2 producing either 0.6 or 1.6 pg P-CTX-1 eq./cell.

**g P-CTX-1 eq. ingested by parrotfish over 30 days**		
	0.6 pg P-CTX-1 eq./*Gambierdiscus*	1.6 pg P-CTX-1 eq./*Gambierdiscus*
30 cm total length parrotfish	1.5×10^{-7}	4.0×10^{-7}
60 cm total length parrotfish	7.1×10^{-7}	1.9×10^{-6}

Table S2. Comparison of CTX ingested by 30 and 60 cm steephead parrotfish (*Chlorurus microrhinos*) feeding for 30 days on turf algae supporting 10 *Gambierdiscus*/cm^2 producing either 0.6 or 1.6 pg P-CTX-1 eq./cell (Table S1) but expressed per g of parrotfish.

**g P-CTX-1 eq./g of fish for parrotfish feeding over 30 days**		
	0.6 pg P-CTX-1 eq./*Gambierdiscus*	1.6 pg P-CTX-1 eq./*Gambierdiscus*
30 cm total length parrotfish, ~0.6 kg [119]	2.5×10^{-10}	6.7×10^{-10}
60 cm total length parrotfish, ~5.0 kg [119]	1.4×10^{-10}	3.8×10^{-10}
